# Supplementary material for: Gender Differences in the Indirect Effect of Psychosocial Work Environment in the Association of Precarious Employment and Chronic Stress: A Cross-Sectional Mediation Analysis
Source: Int J Environ Res Public Health. 2022 Dec 1;19(23):16073. doi: 10.3390/ijerph192316073 (PMC9736292; doi:10.3390/ijerph192316073)
Supplement: Supplementary file 1 [file ijerph-19-16073-s001.zip › ijerph-1969537-supplementary.pdf]

Supplementary Table S1. Goodness-of-ft PRF´s and WLC scales from exploratory factor analysis. Precarious Employment and Stress Study sample, 2020. N men=125, N women= 130.

Variable	Factor 1	Factor 2	Factor 3	Factor 4	Uniqueness
How often do you not have time to complete all your work tasks?	0.28	-0.17	0.55		0.59
Do you have enough time for your work tasks?	0.37		0.53		0.56
Do you have to work very fast?	0.40	0.12	0.43	0.12	0.63
Do you have to deal with other people's personal problems as part of your work?			0.49	-0.12	0.74
Do you work at a high pace throughout the day?	0.35		0.54		0.58
Is your work emotionally demanding?	0.31	0.14	0.65		0.46
Does your work require that you hide your feelings?	0.20	0.26	0.47		0.66
Do you have a large degree of influence on the decisions concerning your work?		0.20	-0.13	0.65	0.52
Do you have any influence on HOW you do your work?		0.17		0.67	0.50
Do you have the possibility of learning new things through your work?	0.12	0.51	-0.32	0.32	0.52
Can you use your skills or expertise in your work?		0.45	-0.30	0.32	0.60
Do you feel that the work you do is important?		0.42		0.13	0.80
How often do you get help and support from your immediate superior, if needed?	0.19	0.65	0.18	0.17	0.48
How often does your immediate superior talk with you about how well you carry out your work?		0.63		0.23	0.55
How often do you get help and support from your colleagues, if needed?		0.64			0.58
Do you feel part of a community at your place of work?		0.63			0.59
Do you feel that your work drains so much of your energy that it has a negative effect on your private life?	0.76	0.12	0.21		0.35
Do you feel that your work takes so much of your time that it has a negative effect on your private life?	0.85		0.10		0.26
Are there times when you need to be at work and at home at the same time?	0.42		0.15	-0.18	0.77
The demands of my work interfere with my private and family life?	0.68		0.16		0.50

Supplementary Table S2. Goodness-of-ft PRF´s and WLC scales from confirmatory factor analysis. Precarious Employment and Stress Study sample, 2020, N men=125, N women= 130.

	χ2 [df], p-value	CFI	ΔCFI	TLI	ΔTLI	RMSEA (90% CI)	ΔRMSEA
Regular CFA							
	732.672 [203]; p<0.001	0.939	-	0.930	-	0.104 (0.096-0.112)	-
Multi-group CFA by women and men							
Configural invariance	1044.355 [406]; p<0.001	0.935	0	0.926	0	0.114 (0.106-0.123)	0.010
Metric invariance	10344.527 [462]; p<0.001	0.930	-0.005	0.924	-0.002	0.116 (0.108-0.124)	0.002
Thresholds invariance	31.217.153 [368]; p<0.001	0.935	0.005	0.938	-0.014	0.105 (0.097-0.113)	-0.011

Supplementary Table S3. Reliability of PRF`s and WLC scales from Cronbach´s alpha. Precarious Employment and Stress Study sample. 2020. N men=125. N women= 130.

Item	Obs	Sign	Item-test correlation	Item-rest correlation	Average interitem covariance	Alpha
Demands						
How often do you not have time to complete all your work tasks?	254	+	0.64	0.48	0.03	0.76
Do you have enough time for your work tasks?	254	+	0.65	0.51	0.03	0.75
Do you have to work very fast?	254	+	0.61	0.48	0.04	0.76
Do you have to deal with other people's personal problems as part of your work?	254	+	0.60	0.41	0.04	0.77
Do you work at a high pace throughout the day?	254	+	0.69	0.57	0.03	0.74
Is your work emotionally demanding?	254	+	0.77	0.64	0.03	0.72
Does your work require that you hide your feelings?	254	+	0.64	0.46	0.03	0.76
Test scale					0.03	0.78
Control						
Do you have a large degree of influence on the decisions concerning your work?	255	+	0.70	0.48	0.03	0.66
Do you have any influence on HOW you do your work?	255	+	0.64	0.43	0.03	0.68
Do you have the possibility of learning new things through your work?	255	+	0.77	0.56	0.02	0.62
Can you use your skills or expertise in your work?	255	+	0.73	0.53	0.02	0.64
Do you feel that the work you do is important?	255	+	0.55	0.36	0.03	0.70
Test scale					0.03	0.71
Support						
How often do you get help and support from your immediate superior. if needed?	246	+	0.81	0.63	0.03	0.66
How often does your immediate superior talk with you about how well you carry out your work?	246	+	0.78	0.56	0.03	0.71
How often do you get help and support from your colleagues. if needed?	246	+	0.73	0.54	0.04	0.72
Do you feel part of a community at your place of work?	246	+	0.72	0.52	0.04	0.72
Test scale					0.03	0.76
Work-life conflict						
Do you feel that your work drains so much of your energy that it has a negative effect on your private life?	253	+	0.85	0.70	0.04	0.72
Do you feel that your work takes so much of your time that it has a negative effect on your private life?	253	+	0.89	0.76	0.04	0.68
Are there times when you need to be at work and at home at the same time?	253	+	0.61	0.40	0.07	0.85
The demands of my work interfere with my private and family life?	253	+	0.81	0.65	0.05	0.74
Test scale					0.05	0.81
